# Application of Amorphous Calcium Phosphate Agents in the Prevention and Treatment of Enamel Demineralization

**DOI:** 10.3389/fbioe.2022.853436

**Published:** 2022-05-13

**Authors:** Jiarong Yan, Hongye Yang, Ting Luo, Fang Hua, Hong He

**Affiliations:** ^1^ The State Key Laboratory Breeding Base of Basic Science of Stomatology (Hubei-MOST) and Key Laboratory of Oral Biomedicine Ministry of Education, School and Hospital of Stomatology, Wuhan University, Wuhan, China; ^2^ Department of Orthodontics, School and Hospital of Stomatology, Wuhan University, Wuhan, China; ^3^ Department of Prosthodontics, School and Hospital of Stomatology, Wuhan University, Wuhan, China; ^4^ Center for Evidence-Based Stomatology, School and Hospital of Stomatology, Wuhan University, Wuhan, China; ^5^ Division of Dentistry, School of Medical Sciences, Faculty of Biology, Medicine and Health, University of Manchester, Manchester, United Kingdom

**Keywords:** enamel, demineralization, remineralization, amorphous calcium phosphate, hydroxyapatite

## Abstract

Enamel demineralization, as a type of frequently-occurring dental problem that affects both the health and aesthetics of patients, is a concern for both dental professionals and patients. The main chemical composition of the enamel, hydroxyapatite, is easy to be dissolved under acid attack, resulting in the occurrence of enamel demineralization. Among agents for the preventing or treatment of enamel demineralization, amorphous calcium phosphate (ACP) has gradually become a focus of research. Based on the nonclassical crystallization theory, ACP can induce the formation of enamel-like hydroxyapatite and thereby achieve enamel remineralization. However, ACP has poor stability and tends to turn into hydroxyapatite in an aqueous solution resulting in the loss of remineralization ability. Therefore, ACP needs to be stabilized in an amorphous state before application. Herein, ACP stabilizers, including amelogenin and its analogs, casein phosphopeptides, polymers like chitosan derivatives, carboxymethylated PAMAM and polyelectrolytes, together with their mechanisms for stabilizing ACP are briefly reviewed. Scientific evidence supporting the remineralization ability of these ACP agents are introduced. Limitations of existing research and further prospects of ACP agents for clinical translation are also discussed.

## Introduction

Enamel demineralization is one of the most common dental problems which could appear as white spot lesions (WSLs) in the early stage and even progress into cavities if effective interventions are not taken in time (Julien et al., 2013). In the normal oral environment, hydroxyapatite on the enamel surface contacts saliva and maintains the balance of dissolution and redeposition (Sollböhmer et al., 1995; Featherstone, 2004), hydroxyapatite could be dissolved into calcium and phosphorus ions while calcium and phosphorus ions in saliva could crystallize directionally and orderly, forming the enamel-like hydroxyapatite structure on the surface of the enamel (Dorozhkin, 1997). When the oral hygiene condition is poor, plaque biofilms form and adhere onto the enamel surface decomposing sugars, producing organic acids, and resulting in an acidic pH environment around the enamel. Under this circumstance, the dissolution-redeposition balance of hydroxyapatite is broken. The dissolution of hydroxyapatite occurs faster than the deposition of calcium and phosphorus ions, which eventually leads to the occurrence of enamel demineralization.

Based on the etiology of enamel demineralization, the strategies to prevent or treat enamel demineralization include: 1) Using antibacterial agents such as mouthwash or toothpaste containing antibacterial drugs (Hefti and Huber, 1987; Afennich et al., 2011; Hossainian et al., 2011; Rösing et al., 2017; Ahmed et al., 2019; Bijle et al., 2019; Guven et al., 2019; Karadağlıoğlu et al., 2019; Shang et al., 2020) which could inhibit the accumulation and adhesion of cariogenic bacteria on the enamel surface to reduce the acid production from plaque biofilms; 2) Using fluorinated agents such as fluoride mouthwash (Chow et al., 2000; Songsiripradubboon et al., 2014; Larsson et al., 2020) and fluoride varnish (Gontijo et al., 2007; Marinho, 2009; Perrini et al., 2016), which could not only inhibit cariogenic bacteria but release fluorine, co-crystallize with calcium and phosphorus ions to form fluorapatite on the enamel surface (Margolis and Murphy, 1986; Zandim-Barcelos et al., 2011).

In addition to the above strategies, the enamel biomimetic remineralization strategy, which bases on the natural enamel crystallization process (Cölfen and Mann, 2003), is being studied extensively due to its biomimetic mineralization capability (Chen et al., 2015; Wang et al., 2017). According to the nonclassical crystallization theory, the crystallization processes of natural enamel could be interpreted as the following steps: 1) Calcium and phosphorus ions aggregating together to form amorphous calcium phosphate (ACP); 2) Amelogenin stabilizing ACP into clusters; 3) ACP then directionally arranging to form bundles of hydroxyapatite, then gradually forming enamel crystal, and finally forming enamel prism (Beniash et al., 2009; Yang et al., 2010; Kwak et al., 2016). To mimic the crystallization process of natural enamel and to achieve remineralization of demineralized enamel, ACP needs to be stabilized and then crystallizes directionally and orderly to form the enamel-like hydroxyapatite. In this review, we mainly focused on how different agents stabilize ACP and their remineralization effects on enamel demineralization.

## Amelogenin and its Analogs

**FIGURE 1:**
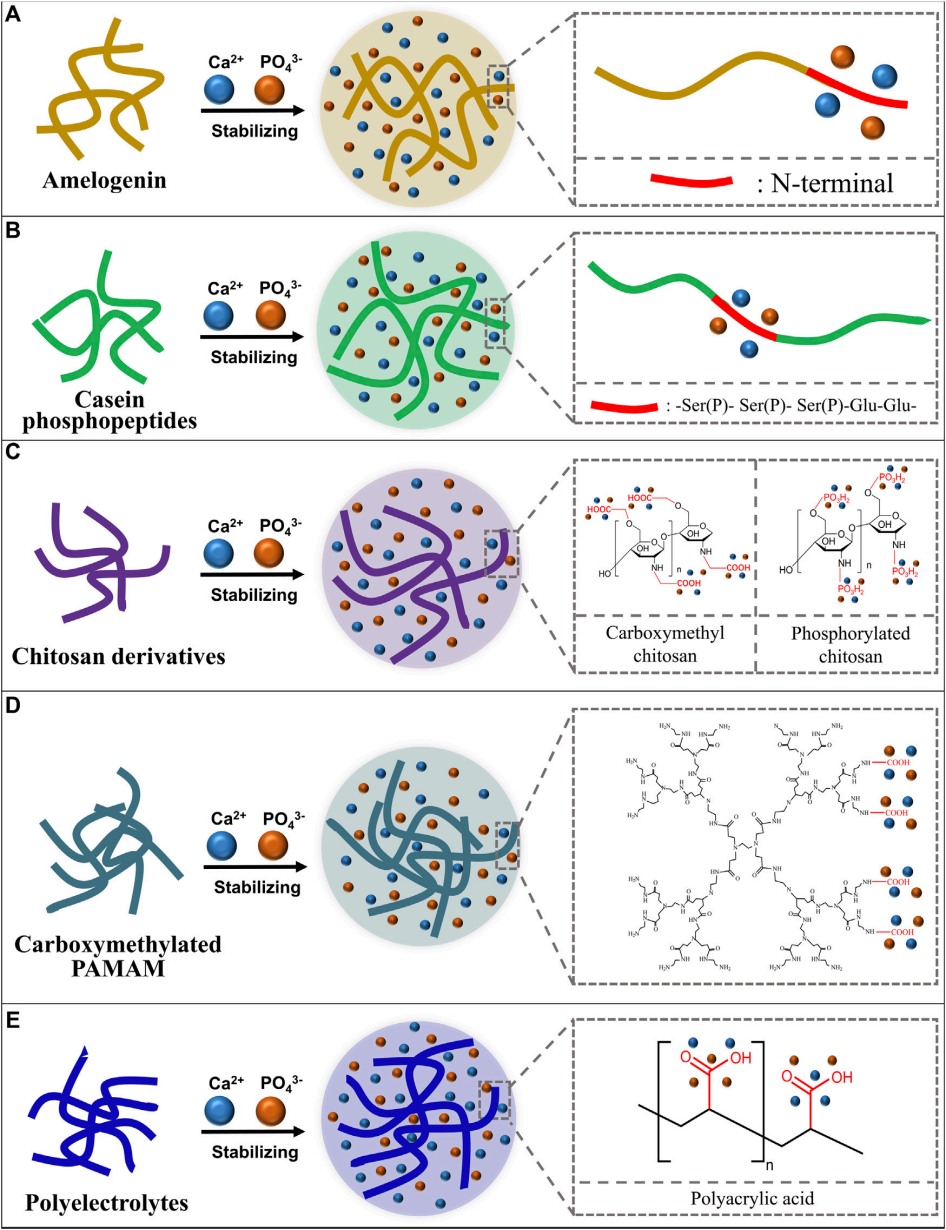
Schematic diagram of ACP stabilizers and how they stabilize the ACP. **(A)** Amelogenin stabilizes calcium and phosphorus ions with its N-terminal; **(B)** Casein phosphopeptides stabilize calcium and phosphorus ions with the -Ser(P)- Ser(P)- Ser(P)-Glu-Glu- sequence; **(C)** Chitosan derivatives stabilize calcium and phosphorus ions with functional groups; **(D)** Carboxymethylated poly-amidoamine (PAMAM) stabilizes calcium and phosphorus ions with carboxyl groups; **(E)** Polyelectrolytes stabilize calcium and phosphorus ions with functional groups.

Amelogenin (Amel) plays an important role in the formation of natural enamel (Wright et al., 2011; Moradian-Oldak, 2012; Ruan and Moradian-Oldak, 2015). Amelogenin could interact with calcium and phosphorus ions through the tyrosine enrichment segment on its N-terminal and stabilize calcium and phosphorus ions to an amorphous state (Figure 1A). The C-terminal of Amel could guide ACP to crystallize into hydroxyapatite directionally (Tsiourvas et al., 2015). There are studies using chitosan to load amelogenin and form the chitosan-amelogenin gel (CS-Amel gel) and using this gel system for reconstruction of demineralized enamel. The CS-Amel gel could stabilize calcium and phosphorus ions into ACP, guide ACP to form the enamel-like crystals which bind closely with natural enamel crystals (Ruan et al., 2013; Ruan et al., 2014). In addition to the direct application of amelogenin, there are studies focused on the remineralization effect of amelogenin analogs. Zhong et al., 2021 self-assembled the N-terminal tyrosine segment of amelogenin to form leucine-rich amelogenin peptide (LRAP) and evaluated the stabilizing and directional guiding abilities of LRAP to calcium and phosphorus ions in mineralizing solutions. LRAP could stabilize calcium and phosphorus ions into ACP effectively and guide ACP to grow along its C-axis into bundles of hydroxyapatite crystals. Wang combined a phase conversion lyase (PTL) which mimics the function of the N-terminal of amelogenin with a synthetic peptide chain which has the function of the C-terminal of amelogenin to form amyloid amelogenin analog (PTL/C-AMG) (Wang Y et al., 2020). The PTL/C-AMG could combine calcium and phosphorus ions to form hydroxyapatite and promote the extension growth of hydroxyapatite crystals on the surface of natural enamel and eventually forms a highly ordered hydroxyapatite structure with mechanical properties similar to that of natural enamel. Lv and colleagues synthesized a short-chain polypeptide (QP5) based on the amino sequence of amelogenin and proved the stabilizing ability of QP5 to calcium and phosphorus ions. They verified the remineralization ability of QP5 to initial enamel demineralization in an *in vitro* enamel demineralization model and further confirmed its remineralization ability and potential for clinical transformation in a rat caries model (Lv et al., 2015; Han et al., 2017).

## Casein Phosphopeptides

Casein phosphopeptides (CPP) are casein extracts from milk which could markedly increase the apparent solubility of calcium phosphate ions by forming ACP (Reeves and Latour, 1958). Researchers found that the main active sequence of CPP, the phosphoserine - glutamate cluster (-Ser(P)- Ser(P)- Ser(P)-Glu-Glu-), could stabilize calcium and phosphate ions and form the CPP stabilized ACP complex (CPP-ACP) (Adamson and Reynolds, 1996) to avoid the spontaneously crystallizing, phase conversing and precipitating of calcium and phosphorus ion (Shen et al., 2001) (Figure 1B). Reynolds soaked artificially demineralized enamel in CPP-ACP solution and found that CPP-ACP could remineralize the subsurface demineralization of enamel effectively. The mechanism may be that CPP can maintain a high concentration of calcium and phosphorus ions in the solution to infiltrate into the subsurface lesion area to achieve efficient enamel remineralization (Reynolds, 1997). The team further validated the preventive effect of CPP-ACP on enamel demineralization in a rat caries model (Reynolds et al., 1995). With the U.S. Food and Drug Administration and other regulatory agencies confirming the biosafety of CPP-ACP (Cochrane et al., 2010), CPP-ACP is added into oral health care products such as Tooth Mousse (GC, Tokyo, Japan) (Rees et al., 2007) and Tooth Mousse Plus (CPP-ACPF, GC, Tokyo, Japan) (Hamba et al., 2011; Bataineh et al., 2017; Olgen et al., 2021). These agents have been gradually used in clinical practice and have been studied in a number of clinical trials (Sitthisettapong et al., 2015; Güçlü et al., 2016; Munjal et al., 2016; Thierens et al., 2019). However, the remineralization ability of CPP-ACP and CPP-ACPF for WSLs remains unknown. Researchers suggested that CPP-ACP and CPP-ACPF may have the ability to prevent and treat WSLs, but their effects are not significantly greater than using fluoride agent alone (Pithon et al., 2019; Wang D et al., 2020). In addition, casein related allergy in certain populations also limits the clinical use of CPP-ACP and CPP-ACPF.

## Polymers

In addition to the aforementioned amelogenin and its analogs and CPP, some kinds of polymers can also stabilize calcium and phosphate ions, including chitosan derivatives, poly-amidoamine and polyelectrolytes.

### Chitosan Derivatives

Chitosan derivatives, such as carboxymethyl chitosan (CMC) and phosphorylated chitosan (Pchi) could bind calcium ions through chelation reaction of carboxyl groups and calcium ions and then bind phosphate ions to form ACP (Figure 1C). The recrystallization of demineralization enamel is realized by the ordered crystallization of ACP to form enamel-like hydroxyapatite crystals (Zhang et al., 2014; Zhang et al., 2018). Zhu combined carboxymethyl chitosan (CMC) and lysozyme (LYZ) to stabilize ACP and formed the CMC/LYZ-ACP nano-gel, which can regenerate prism-like remineralized enamel layer on the surface of eroded enamel to realize the remineralization (Zhu et al., 2021). Song successively added CaCl_2_ and K_2_HPO_4_ into Pchi solution to construct the Pchi-ACP nano-complex. X-ray diffraction and selective electron diffraction results confirmed the amorphous state of the nano-complex. And the results of scanning electron microscopy and micro-CT proved that the Pchi-ACP nano-complex could realize the remineralization of demineralized enamel (Song et al., 2021).

### Poly-Amidoamine

Poly-amidoamine (PAMAM) was first synthesized by Tomalia in the 1980s (Tomalia et al., 1985). PAMAM contains a large number of amide groups that have the similar function to peptide bonds so that PAMAM could simulate functions of a variety of proteins and peptides (Svenson and Tomalia, 2012). PAMAM can have mineralization property through the modification of carboxyl groups. The carboxyl-modified PAMAM (PAMAM-COOH) could combine calcium ions through carboxyl groups and further attract phosphate ions to stabilize calcium and phosphate ions into ACP (Khopade et al., 2002; Zhou et al., 2007; Zhou et al., 2013) (Figure 1D). ACP could form enamel-like hydroxyapatite orderly on the surface of demineralized enamel through the crystallization guidance of PAMAM-COOH (Chen et al., 2013). Another study found that PAMAM-COOH can induce calcium and phosphorus ions to grow and crystallize along the *z*-axis on the surface of demineralized enamel, and the microhardness of the remineralized enamel is comparable to that of the natural enamel (Chen M et al., 2014).

### Polyelectrolytes

Polyelectrolytes are a class of polymorphs with ionizable units, which could ionize into charged polymorphs and counter-ions with opposite charge in aqueous solution (Koetz and Kosmella, 2007) such as polyacrylic acid (PAA), polyallylamine (PAH), polyaspartic acid (PASP), et al. PAA has rich carboxyl groups to combine with calcium ions to form the -COO^-^/Ca^2+^ structure (Huang et al., 2008), so that PAA can stabilize ACP (Gower, 2008; Dey et al., 2010) (Figure 1E). Qi added calcium and phosphorus ions into the PAA solution to construct the PAA-ACP complex and verified the stability of the PAA-ACP complex by solution turbidity analysis and dynamic light scattering. Scanning electron microscopy, transmission electron microscopy, infrared spectroscopy and X-ray diffraction analyses proved the remineralization ability of PAA-ACP (Qi et al., 2018). Our group used PAA to stabilize amorphous calcium phosphate, and then loaded PAA-ACP with aminoated mesoporous silicon nanoparticle (aMSN) to form the PAA-ACP@aMSN delivery system. The PAA-ACP@aMSN was proved to have the ability to promote enamel remineralization and surface microhardness analysis and X-ray diffraction analysis showed that the remineralization layer induced by PAA-ACP@aMSN had comparable mechanical property and crystal texture to natural enamel (Hua et al., 2020). PAA-ACP could also act as a dental adhesive filler to endow adhesives with enamel remineralization ability (Wang et al., 2018). Other polyelectrolytes like polyallylamine (Niu et al., 2017; Yang et al., 2017), polyaspartic acid (Zhou, et al., 2021), polyglutamic acid (Sikirić, et al., 2009; Terauchi, et al., 2019) could alsostabilize calcium and phosphorus ions but the treatment or prevention effect of these polyelectrolyte-stabilized ACPs for enamel demineralization remains to be further investigated.

## ACP Particles

As a kind of amorphous substance, ACP is easy to spontaneously transform into apatite crystal in an aqueous solution from the thermodynamics point of view (Eanes et al., 1965; Chow et al., 1998). Therefore, in addition to the application of stabilizers to stabilize ACP in an amorphous state, another way to stabilize ACP is to store the prepared ACP in an anhydrous dry granular state to form ACP particles. Since the 1990s, ACP particle has been gradually used as a bioactive additive in the studies of tooth remineralization (Skrtic and Eanes, 1996). ACP particle could act as a bioactive filler of dental filling resin to endow the filling resin with the ability of continuous releasing of calcium and phosphorus ions to promote the formation of hydroxyapatite (Skrtic et al., 2004). However, the uncontrollable agglomeration of ACP particles in the resin affects the mechanical properties of resin such as bonding strength and bending strength, so that ACP particles are only suitable for materials with low requirements on mechanical properties, such as pit and fissure sealant (Skrtic et al., 2004; Dunn, 2007). In 2011, Xu synthesized nano ACP (NACP) by spray drying method for the first time and mixed it into dental resin as filler. The NACP modified dental resin could release calcium and phosphorus ions in an acidic environment, and the mechanical properties of the resin are even better than commercial dental resin materials (Xu et al., 2011). Since then, a large number of studies added NACP to dental materials such as orthodontic bonding resins, sealants, resin-modified glass ions and other materials, and verified their calcium and phosphorus ion release ability and enamel remineralization ability (Chen C et al., 2014; Ma et al., 2017; Liu et al., 2018; Xie et al., 2019; Gao et al., 2020; Ibrahim et al., 2020).

## ACP Agents Versus Other Remineralization Agents

In addition to ACP agents, there are many other enamel remineralization agents such as fluorine containing agents, hydroxyapatite preparations and tricalcium phosphate. *In vitro* and *in vivo* studies have been conducted to compare the remineralization performance of ACP agents and other agents (Table 1). However, the conclusion varied among these studies. Some studies found that ACP agents have better remineralization effect than other agents, while others suggested that the remineralization effect of ACP agents is similar to or no better than other agents. Whether the ACP agents have better remineralization properties than other agents needs to be further investigated in the future research.

**TABLE 1:** Comparison of the remineralization performance of ACP agents with other products.

Authors	Study type	Agents	Interventions	Evaluation methods	Results	Conclusions
Aras et al. (2020)	*In vitro*	Fluoride gel, CPP-ACP, CPP-ACPF, NovaMin-Fluoride, Xylitol-HAP-Fluoride, Ozone-Fluoride	Following manufacturer’s instructions for 30 days	DIAGNOdent	There were significant differences in the scores before and after the remineralization procedure in all experimental groups. CPP-ACPF provided significantly more remineralization than other experimental groups.	Remineralization of demineralized areas was achieved in all experimental groups. The highest degree of remineralization according to the DIAGNOdent scores before and after the procedure was observed in the CPP-ACPF group.
Farzanegan et al. (2018)	*In vitro*	0.05% NaF and 0.05% ACP solution	1 min/day for 10 weeks	Microhardness tester	Microhardness of samples in NaF and ACP groups both had significantly improved after the treatment. No significant differences were found neither between the fluoride and ACP, nor the fluoride and control groups.	Both 0.05% NaF solution and 0.05% ACP solution enhanced the enamel microhardness ad are suitable for treatment of white spot lesions.
Farzanegan et al. (2019)	*In vitro*	0.05% ACP, 0.5% ACP and 0.05% fluoride solutions	1 min/day for 10 weeks	Colorimeter	There was no significant difference among 0.05% ACP, 0.5% ACP and 0.05% fluoride solutions. And a significant different was noted between these solutions and distilled water.	ACP is as effective as fluoride in the color improvement of WSLs.
(Oliveira et al. (2014)	*In vitro*	CPP-ACP, CPP-ACPF, 1.1% NaF dentifrice	Following manufacturer’s instructions for 30 days	QLF	1.1% NaF dentifrice showed greater remineralization than CPP-ACP and CPP-ACPF.	The 1.1% NaF dentifrice demonstrated overall greater remineralization ability.
Behrouzi et al. (2020)	*In vitro*	CPP-ACPF, Remin Pro	Following manufacturer’s instructions for 20 weeks	Microhardness tester	The hardness of samples in CPP-ACPF and Remin Pro groups significantly in-creased, but there was no statistic difference between CPP-ACPF and Remin Pro groups.	CPP-ACPF and Remin Pro can efficiently increase the hardness of incipient enamel lesions.
Yadav et al. (2019)	*In vitro*	Bio-minF, CPP-ACPF	Twice a day, 4 min per time for 6 weeks	Spectrophotometer, DIAGNOdent	Bio-minF, and CPP-ACPF showed significant recovery of color change and fluorescence loss. Bio-minF had higher fluorescence recovery value than CPP-ACPF and showed similar color change value to CPP-ACPF.	Both Bio-minF and CPP-ACPF could remineralize artificial enamel caries and showed improvement in color change and fluorescence loss.
Kamath et al. (2017)	*In vitro*	Nano-HA, CPP-ACPF, TCP	3 min/day for 14 days	DIAGNOdent, SEM, EDX	SEM evaluation showed favorable surface change in all groups. DIAGNOdent and EDX readings was statistically nonsignificant among groups.	All agents showed comparable remineralization potential.
Tahmasbi et al. (2019)	*In vitro*	0.05% NaF, CP-ACPF, Remin Pro paste	Once a day, 5 min per time for 14 days	Microhardness tester	0.05% NaF was more efficient than Remin Pro and CPP-ACPF. Remin Pro and CPP-ACPF were not significantly difference from the control group.	NaF mouthwash had the greatest efficacy for prevention of enamel demineralization compared with Remin Pro and CPP-ACPF.
Jo et al. (2014)	*In vitro*	1000ppm F, CPP-ACP,and fTCP containing toothpaste	Twice a day for 2 weeks	QLF-D Biluminator	Fluorescence greatly increased in the fTCP and CPP-ACP groups compared with the fluoride and control groups.	fTCP and CPP-ACP seem to be more effective in reducing WSLs than 1000 ppm F containing toothpastes.
Bhadoria et al. (2020)	*In vitro*	CPP-ACPF, fTCP	Twice a day, 2 min per time for 10 days	Microhardness tester	fTCP shows significantly higher increase in mean microhardness when compared to CPP-ACPF and control group.	f-TCP showed comparatively more remineralization potential than CPP-ACPF.
Brochner et al. (2011)	RCT	CPP-ACP and fluoride containing toothpaste	Once a day for 4 weeks	QLF	A statistically significant regression of the WSL was disclosed in both study groups compared to baseline, but there was no difference between the groups.	The application of CPP-ACP could resulte in a reduced area of the lesions after 4 weeks but the improvement was however not superior to “natural” regression with daily use of fluoride toothpaste.
Huang et al, (2013)	RCT	CPP-ACPF and PreviDent fluoride varnish	CPP-ACPF group: twice a day for 8 weeks; Varnish group: a single application at the start of the study.	Visual assessment	The mean improvements assessed by the professional panel were 21%, 29%, and 27% in the CPP-ACPF, fluoride varnish, and control groups, respectively.	CPP-ACPF and PreviDent fluoride varnish do not appear to be more effective than normal home care for improving the appearance of white spot lesions over an 8-week period.
Akin and Basciftci, (2020)	Clinical controlled trial	0.025% NaF rinse and CPP-ACP	Following manufacturer’s instructions after brushing teeth with F containing toothpaste for 6 months	Image processing with AutoCAD for quantitative analysis	The area of the white spot lesions decreased significantly in all groups. The success rate of CPP-ACP was significantly higher than that of NaF.	The use of CPP-ACP can be more beneficial than fluoride rinse for postorthodontic Remineralization.
Singh et al. (2016)	RCT	Fluoride toothpaste, fluoride varnish with fluoride toothpaste, CPP-ACP with fluoride toothpaste,	Subjects were advised to brush twice daily with fluoride toothpaste for 1, 3,6 months.	DIAGNOdent, Visual assessment	The mean visual and DIAGNOdent scores at various time intervals of observations were decreased more when fluoride varnish and CPP-ACP were used in addition to daily use of fluoride toothpaste, but the differences were not statistically significant.	The use of fluoride varnish and CPP-ACP in addition to twice daily use of fluoride toothpaste had no additional benefit in the remineralization of post-orthodontic WSLs.
			varnish application: a single application at the start of the study.			
			CPP-ACP application: twice daily after brushing teeth			

CPP-ACP, casein phosphopeptide- amorphic calcium phosphate; CPP-ACPF, casein phosphopeptide- amorphic calcium phosphate with fluoride; HAP, hydroxyapatite; QLF, quantitative light-induced fluorescence; TCP, tricalcium phosphate; SEM, scanning electron microscope; EDX, energy dispersive X-ray; fTCP, fluoride tricalcium phosphate; RCT, randomized controlled trial.

## Discussion

ACP agents have outstanding preventive and therapeutic capacity to enamel demineralization due to their ability to form the enamel-like hydroxyapatite on the surface of demineralized enamel (Kwak et al., 2016). However, Since ACP is easy to agglomerate and is unstable in an aqueous solution (Chow et al., 1998), the main challenge in applying the ACP for enamel remineralization is its stabilization. Many different materials that could stabilize calcium and phosphorus ions, including amelogenin and its analogs (Tsiourvas et al., 2015; Wang Y et al., 2020), casein phosphopeptides (Cross et al., 2005), polymers like chitosan derivatives (Zhu et al., 2021), carboxymethylated PAMAM (Chen et al., 2013) and polyelectrolytes (Hua et al., 2020), have been used in studies to stabilize calcium and phosphorus ions into ACP. Another strategy is to store the ACP in a water-free state so that ACP particles and NACP particles are formed (Betts et al., 1975; Xu et al., 2011). The remineralization abilities of these ACP agents have been confirmed in previous studies. However, except for CPP-ACP and CPP-ACPF which has been commercialized (Reise et al., 2021), most of the other ACP agents are still at *in vitro* experimental stage. Tt is still uncertain whether these ACP agents can achieve the remineralization of demineralized enamel *in vivo*. In addition, most of the studies evaluated the remineralization ability of ACP agents by measuring the hardness recovery of demineralized enamel (Gokkaya et al., 2020), observing the mineral deposition on demineralized enamel (Hua et al., 2020), or measuring the lesion depth (Soares-Yoshikawa et al., 2021). None of the above-mentioned evaluation methods can directly confirm whether ACP agents could form the enamel-like hydroxyapatite. The biomimetic remineralization ability of ACP agents needs further investigation. To further promote the translation of ACP agents into clinical application, basic studies with adequate evaluation methods as well as relevant *in vivo* studies are still needed. In addition, whether ACP agents have better remineralization effects compared to other agents remains to be further explored.

ACP complexes are in the amorphous state of the liquid phase (Chen et al., 2013; Niu et al., 2017; Qi et al., 2018; Song et al., 2021), and ACP particles (Skrtic et al., 2004; Xu et al., 2011) are solid powders. Neither the liquid nor the solid form is convenient for storage and direct application in the oral environment. Studies has been conducted to address the storage and application challenges of ACP agents:

1) Mouthwash. Studies used carriers like chitosan (Ruan et al., 2013) and carboxymethyl chitosan (Zhu et al., 2021) to load ACP agents and these delivery systems can be applied in the oral environment in the form of mouthwash.
2) Toothpaste and tooth desensitizer. Another form of application of the ACP agents is to make them into pastes. Our group used mesoporous silicon nanoparticles to load ACP agents to achieve the enrichment and storage of ACP and this delivery system can be applied as the filler of toothpaste (Hua et al., 2020). CPP-ACP agents can be used as desensitizers in the form of pastes (Pei et al., 2013; Chandavarkar and Ram, 2015; Yang et al., 2018).
3) Resin product. Particulate forms of ACP have been incorporated into resin products, like adhesives (Wang et al., 2018), pit and fissure sealants (Utneja et al., 2018), varnishes (Schemehorn et al., 2011) to achieve convenient applications that do not depend on patient compliance.

Mouthwash form of the ACP agent is convenient to use, but the relatively low concentration of ACP and its inability to persist on the enamel surface for long periods lead to the limited effectiveness of ACP agents to enamel remineralization. The paste-like application form could effectively enhance the concentration of ACP and could maintain a high concentration of ACP on the enamel surface during application, but like mouthwash, it still has a short duration of hydroxyapatite formation due to the effect of saliva flushing. ACP agents modified resin products can release ACP on the enamel surface for a long period thus achieving the long-term prevention or treatment of enamel demineralization. However, the effect of ACP agent incorporation on the performance of these products like mechanical performance and biocompatibility needs further exploration. And the long-term stability of the ACP release from these products should be considered in future studies.

## Conclusion

Herein we summarize the strategies of stabilizing ACP. Calcium and phosphorus ions can be stabilized to the ACP state using a variety of methods, but the preventive and therapeutic effects of these ACP agents on enamel demineralization still await further investigation. There are three main forms of storage and application of ACP agents, namely mouthwash, toothpaste/tooth desensitizer, resin product. However, due to the shortcomings of the above-mentioned forms of ACP agents, more easy-to-use and long-lasting forms of ACP agents remain to be further explored.
